# Effects of Broiler Breeder Age on Egg Quality, Incubation Performance, and Early Progeny Physiological and Muscle Fiber Development

**DOI:** 10.1111/jpn.70071

**Published:** 2026-05-07

**Authors:** Helder Freitas de Oliveira, Alessandra Gimenez Mascarenhas, José Henrique Stringhini, Nadja Susana Mogyca Leandro, Marcos Barcellos Cafe, Emmanuel Arnhold, Raíssa Monteiro de Alvarenga, Ana Caroline Romão Silva, Jean Kaique Valentim, Heloisa Helena de Carvalho Mello

**Affiliations:** ^1^ Department of Animal Science Federal University of Goiás (UFG) Goiânia Goiás Brazil; ^2^ Department of Animal Production Federal Rural University of Rio de Janeiro (UFRRJ) Seropédica Rio de Janeiro Brazil

**Keywords:** embryonic development, incubation efficiency, metabolism, muscle fibers

## Abstract

This study evaluated the effects of breeder hen age on the incubation performance, embryonic development, and progeny quality of Cobb500 broiler breeders at 39, 51, and 69 weeks of age. A total of 870 eggs were collected, of which 810 were incubated; these were assigned to a completely randomized design with three treatments and nine replicates of 30 eggs. The average egg weights were 64.68, 69.77, and 72.40 g for breeders aged 39, 51, and 69 weeks, respectively. The incubation traits, chick quality at hatch, and embryonic development indicators, including relative embryo and liver weights, eggshell calcium content, serum glucose, and breast muscle fiber number, were evaluated. Eggs from 69‐week‐old breeders were heavier and resulted in heavier chicks at hatch and placement (*p* < 0.001), with lower dehydration than did chicks from 39‐week‐old breeders (*p* = 0.001). Hatchability, hatchability of fertile eggs, and hatch window were not affected by breeder age (*p* > 0.05). Embryonic mortality during Phase I was greater in eggs from 69‐week‐old breeders (*p* = 0.007). Embryos from younger breeders (39 weeks) presented greater relative developmental efficiency at 6 and 13 days of incubation (*p* ≤ 0.015). Progeny from older breeders (51 and 69 weeks) was associated with a greater incidence of physical quality scores between 86 and 90 (*p* = 0.050) and a greater number of breast muscle fibers in female chicks (*p* = 0.006). Eggshell calcium content, serum glucose concentration, and relative liver weight were not influenced by breeder age (*p* > 0.05). Breeder age significantly influences egg quality, embryonic development, and progeny characteristics, and advanced breeder age represents a trade‐off between improved chick size and quality and increased early embryonic mortality.

## 1 Introduction

Broiler production fundamentally depends on the quality of fertile eggs and chick output, with incubation representing a critical process in this context. For production efficiency, day‐old chicks must exhibit high physical, sanitary, and immunological qualities (Araújo et al. 2019). Chick quality at hatch is strongly associated with broiler performance throughout the production cycle, directly influencing the productivity of the poultry industry (Decuypere et al. 2001). In this context, appropriate management of heavy breeder hens, combined with continuous monitoring of incubation parameters, plays a key role in ensuring the production of high‐quality chicks (Nowaczewski et al. 2016).

Several parameters, including body weight, the Tona score, and yolk sac retraction, which are essential indicators of production success, have been identified as predictors of chick performance (Tona et al. 2003; Goliomytis et al. 2015). According to Ulmer‐Franco et al. (2010), high‐quality chicks should weigh between 40 and 44 g, present a well‐healed navel, dry down feathers, and display activity at hatch. The identification and evaluation of these parameters are therefore crucial for assessing chick quality and, consequently, incubation efficiency.

Among the factors that influence egg and chick quality, breeder age is a major determinant of reproductive and developmental outcomes, affecting egg characteristics, embryonic metabolism, and progeny development, particularly in modern broiler strains (Fernandes et al. 2014; Nangsuay et al. 2016; Crosara et al. 2019). Breeder age also alters the relationship between egg weight and chick weight at hatch, with eggs from older hens producing heavier chicks (Zocche et al. 2016).

In addition to genetic factors, egg quality is influenced by nutrient availability, which is essential for chick growth and health. Egg nutritional quality may vary with breeder age and can consequently affect chick quality and incubation parameters (Tona et al. 2004). An adequate nutrient supply, particularly of lipids and calcium, is critical for embryonic development and posthatch growth (Ulmer‐Franco et al. 2010; Xavier et al. 2021). In addition to genetic factors, breeder age affects both the nutritional composition of the egg and the structural properties of the shell, influencing gas exchange, nutrient allocation, and embryo growth (Araújo et al. 2017; Hamidu et al. 2007). Younger breeders often produce eggs with thicker shells, which may limit oxygen diffusion and slow early development.

These breeder‑age effects directly intersect with key physiological events during embryogenesis, including the transition from anaerobic to aerobic metabolism and the development of organs such as the liver and skeletal muscle, which influence early chick growth potential (Moran 2007; Maiorka et al. 2016).

Although the effects of breeder age on individual traits such as egg weight or hatchability are well documented, most studies have evaluated these outcomes in isolation or focused on limited developmental endpoints. In contrast, fewer studies have simultaneously assessed egg quality, incubation responses, and early progeny development, with particular emphasis on embryonic growth, metabolic indicators, liver development, and breast muscle fiber characteristics, within a single experimental framework. Therefore, an integrated evaluation of these parameters may contribute to a more comprehensive understanding of the biological implications of breeder age under practical production conditions.

We hypothesised that breeder age influences egg quality and incubation responses and is associated with differences in early physiological and morphological development of broiler progeny during embryonic and immediate post‑hatch stages. Accordingly, this study evaluated the effects of breeder age on egg quality, incubation responses, and early progeny development, with an emphasis on embryonic growth, metabolic indicators, liver development, and breast muscle fiber characteristics.

## 2 Materials and Methods

### 2.1 Location and Ethical Approval

The experiment was conducted at the Experimental Poultry Facility of the Poultry Science Sector, Department of Animal Science, School of Veterinary Medicine and Animal Science, Federal University of Goiás (UFG), Goiânia, Goiás, Brazil. The study was approved by the Ethics Committee on the Use of Animals (CEUA) of UFG under protocol No. 076/18.

### 2.2 Experimental Design

This study was conducted under commercial conditions using Cobb 500 breeder hens at 39, 51, and 69 weeks of age from the same commercial farm, which was managed under standardised environmental and nutritional programs according to the strain guidelines. Eggs were obtained from a commercial hatchery and had an average storage period of 3 to 5 days before incubation, which is considered acceptable for hatchable eggs. During storage, eggs were maintained under controlled conditions recommended for commercial broiler breeder operations. Prior to incubation, eggs were sanitized according to routine hatchery procedures.

A total of 870 hatchable eggs were collected and assigned to a completely randomized design (CRD) with three treatments (breeder ages) and nine replicates of 30 eggs each. The average egg weights were 64.68 ± 0.57 g, 69.77 ± 0.58 g, and 72.40 ± 0.58 g for eggs from breeders aged 39, 51, and 69 weeks, respectively. For egg quality analysis, 60 eggs were randomly selected (20 per treatment). The remaining 810 eggs were incubated. Single‐stage incubators (model MA01DA – Gaiolas Almeida, Aparecida de Goiânia, GO, Brazil) with a capacity of 270 eggs were used, each equipped with three incubation trays. Eggs from all breeders were randomly distributed across incubators and trays so that each incubator contained eggs from all the treatments, minimizing potential incubator and tray effects. For incubation and early development variables, the individual egg was considered the experimental unit, with eggs treated as independent observational units due to their random distribution across incubators and trays. Which functioned as both incubator and hatcher. For the hatching phase, incubation trays were replaced with hatching trays at 456 h of incubation (19 days), and temperature and relative humidity were adjusted to hatcher conditions. During this phase, eggs were individually placed in mesh bags to preserve eggshell identification markings and to allow recovery of eggshell fragments for subsequent calcium analysis.

### 2.3 Egg Selection, Preparation, and Quality Assessment

Eggs were visually selected to ensure that only high‐quality eggs were incubated. Dirty, pointed, cracked, broken, or misshapen eggs were discarded. Each egg was individually identified and weighed via a precision digital scale (±0.001 g). After selection, the eggs were distributed into incubation trays, with 90 eggs per tray (30 eggs from each treatment).

Egg quality analysis included measurements of egg, yolk, albumen, and shell weights, as well as yolk and albumen height and diameter (mm). The specific gravity (SG) was determined according to the methods described by Hamilton (1982), where eggs were immersed in saline solutions with densities ranging from 1.050 to 1.100 g/cm^3^ with an interval of 0.005.

Haugh units (HUs) were calculated via the following equation:

$$\mathrm{HU}=100\times \log(H-1.7\times P^{0.37}+7.57)$$

where *H* is the albumen height (mm) and *P* is the egg weight (g) (Silva 2004).

The yolk index was calculated as the ratio of yolk height to yolk diameter, and the albumen index was calculated as the ratio of albumen height to mean albumen diameter. After the measurements, the internal egg contents were separated and weighed individually according to the methods described by Onbasilar et al. (2018). Eggshells were washed, air‐dried at room temperature for 72 h, and weighed. Shell thickness was measured at two points in the equatorial region via a digital caliper, and the mean value was used for statistical analysis (Arslan and Yamak 2020).

### 2.4 Incubation Procedures and Embryonic Parameter Assessment

Eggs were incubated at 37.5°C and a relative humidity (RH) of 60%. Egg turning was programmed to occur every 2 h during the first 456 h of incubation (19 days). Eggs were identified via Arabic numerals marked with a pencil at two points on the eggshell. Eggs were candled on day 7 of incubation to identify infertile eggs and early embryonic mortality.

On incubation days 6, 13, and 18, 20 eggs per treatment were opened to assess embryonic development. Egg contents were transferred to Petri dishes, and the embryos were carefully removed via surgical forceps. Embryos at days 6, 13 and 18 were weighed to determine absolute and relative embryo weight. Embryos at days 13 and 18 were further subjected to necropsy to determine relative liver weight, defined as the ratio between liver weight and embryo weight. At each embryonic sampling time (days 6, 13, and 18), eggs were removed homogeneously across all incubators and trays within each treatment to ensure equivalent incubation conditions and to avoid localized depletion effects.

After 456 h of incubation (19 days), the eggs were transferred to hatching trays, which were set to hatcher conditions at 36.7°C and 70% RH. The hatching process was monitored, and the hatch percentage was recorded from the moment the first chick hatched at 6 h intervals.

### 2.5 Blood Glucose and Muscle Fiber Analysis

During the hatch window (468–504 h of incubation), four chicks per treatment were randomly selected at 6 h intervals for blood collection and glucose determination. Blood samples were collected via cardiac puncture, with euthanasia occurring during the sampling procedure, centrifuged at 3,000 rpm, and stored frozen until analysis via a commercial enzymatic kit. Sampling time was considered an additional factor in the statistical analysis of serum glucose.

For breast muscle fiber analysis, six newly hatched chicks (three males and three females) per treatment were euthanized after 504 h of incubation. Chicks were randomly selected from different trays to minimize potential tray effects. Sex was determined at hatch by wing‐feather examination. Samples of the *pectoralis major* muscle were collected, fixed in formaldehyde solution, processed, and stained with hematoxylin and eosin according to Luna (1968). Histological sections were digitised using an optical microscope. For each animal, ten non‑overlapping microscopic fields were analyzed per section. Muscle fibers were counted and measured using AxioVision 3.0 software following the methodology described by Dubowitz and Brooke (1984).

### 2.6 Eggshell Calcium Content Analysis

Six eggshells were randomly collected at four time points: at the beginning of incubation, on days 6, 13, and 18 of incubation, and at hatch (21 days). Eggshells were predried at 55°C ± 5°C, and the mineral matter content was determined via calcination in a muffle furnace at 600°C. The calcium content was analysed via atomic absorption spectrophotometry according to the method described by Silva and Queiroz (2002).

### 2.7 Hatchability and Physical Quality Parameters

Hatching data were recorded, and hatch percentage, infertility, and hatch of fertile eggs were calculated. Chicks' physical quality was assessed based on hatch weight, placement weight, dehydration, and physical quality score. Placement weight was defined as the chick's body weight measured at the time of placement, following the hatch window. The quality score was determined according to the criteria proposed by Tona et al. (2003). Dehydration was calculated as the difference between chick weight at hatch and at placement. The egg‐chick weight ratio was calculated using chick body weight without the residual yolk sac, determined in a subset of chicks used for physical quality assessment.

After the hatch window, all unhatched eggs were opened for embryodiagnosis. Embryonic mortality was classified according to the stage of development: early (0–7 d), middle (8–17 d), and late (18–21 d) embryonic mortality, and the number of infertile eggs and pipped but unhatched chicks was recorded for each treatment.

### 2.8 Statistical Analysis

The data were analyzed via analysis of variance (ANOVA). For most variables, one‐way ANOVA was applied considering breeder age as the fixed effect, followed by Tukey's test for multiple comparisons among means. For the serum glucose concentration, the data were analyzed via two‐way ANOVA considering breeder age (39, 51, and 69 weeks) and sampling time after hatching (0, 12, 24, and 36 h) as fixed effects, including their interaction.

Prior to analysis, the assumptions of ANOVA were evaluated. The normality of the residuals was assessed via the Shapiro–Wilk test, and the homogeneity of variances was verified via Levene's test. When necessary, residual plots were inspected to confirm model adequacy. Variables that did not meet ANOVA assumptions were analyzed via the Kruskal–Wallis nonparametric test. Statistical significance was declared at *p* < 0.05. All analyses were performed via R software (R Core Team 2021). When appropriate, regression analyses were performed to evaluate the relationships between breeder age and the evaluated variables, and the best‐fitting model was selected on the basis of the significance of the regression coefficients and the coefficient of determination (*R* ^2^).

The statistical model used for most variables was as follows:

$$Y_{ij}=\mu +A_{i}+\varepsilon_{ij}$$

where *Y_ij_* is the observed value of the response variable, *µ* is the overall mean, *A_i_* is the fixed effect of breeder age (39, 51, and 69 weeks), and *Ɛ_ij_* is the random error associated with individual egg, which was considered the experimental unit for incubation and early development variables.

For the serum glucose concentration, the following model was applied:

$$Y_{ijk}=\mu +A_{i}+T_{j}+{\left(AT\right)}_{ij}+\varepsilon_{ijk}$$

where *Yᵢⱼₖ* is the observed serum glucose value, *μ* is the overall mean, *Aᵢ* is the fixed effect of breeder age (*i* = 39, 51, 69 weeks), *Tⱼ* is the fixed effect of sampling time (*j* = 0, 12, 24, 36 h), (*AT*)*ᵢⱼ* is the interaction between breeder age and sampling time, and *εᵢⱼₖ* is the random error.

## 3 Results

Breeder age significantly affected (*p* < 0.05) the characteristics of hatchable eggs. Eggs from 69‑week‑old breeders were heavier and presented greater egg weights, yolk percentages, and albumen weights than those from 39‑week‑old breeders did, although the albumen percentage was greater in eggs from younger breeders. Breeder age did not affect (*p* > 0.05) eggshell weight; however, older breeders produced eggs with a lower shell percentage. With respect to eggshell thickness and internal egg quality, eggs from 39‑week‑old breeders presented greater shell thickness, greater yolk index values, and greater specific gravities. No significant differences were detected between eggs from 39‑ and 51‑week‑old breeders, whereas eggs from 69‑week‑old breeders presented lower yolk indices and specific gravity values. Breeder age had no significant effect (*p* > 0.05) on the Haugh unit or albumen index (Table 1).

**Table 1:** Egg composition and quality traits of eggs from heavy breeder hens of different ages.

Variable	Breeder age (weeks)			*p* value	SEM
	39	51	69		
Egg weight (g)	64.68^c^	69.77^b^	72.40^a^	< 0.001	0.585
Yolk weight (g)	18.33^c^	21.18^b^	23.32^a^	< 0.001	0.362
Shell weight (g)	5.60	5.88	5.68	0.194	0.138
Albumen weight (g)	40.75^b^	42.17^ab^	42.99^a^	0.034	0.598
Yolk (%)	28.35^c^	30.65^b^	32.47^a^	< 0.001	0.477
Shell (%)	8.65^a^	8.50^a^	7.68^b^	0.001	0.191
Albumen (%)	62.99^a^	60.84^b^	59.85^b^	< 0.001	0.487
Shell thickness (mm)	0.29^a^	0.25^b^	0.24^b^	< 0.001	0.007
Haugh unit (HU)	69.42	72.97	69.15	0.377	2.132
Yolk index	0.40^a^	0.38^ab^	0.37^b^	0.018	0.006
Albumen index	0.06	0.07	0.07	0.436	0.004
Specific gravity (g/cm^3^)	1.077^a^	1.076^ab^	1.073^b^	0.015	0.001

*Note:* Means followed by different superscript letters within a row differ by Tukey's test (*p* < 0.05).

Abbreviation: SEM, standard error of the mean.

**Figure 1:**
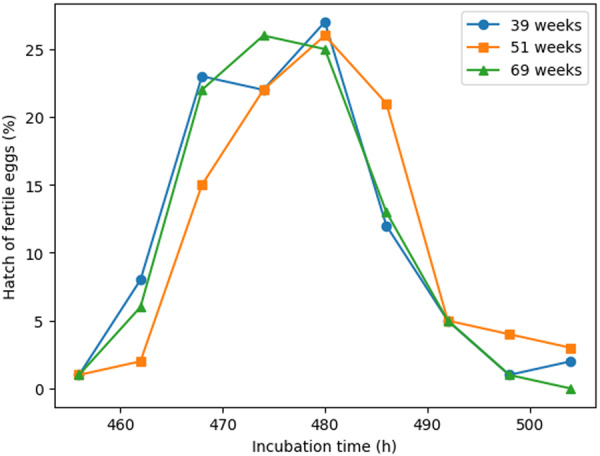
Hatch windows of broiler chicks from heavybred hens of different ages. [Color figure can be viewed at wileyonlinelibrary.com]

Breeder age did not affect (*p* > 0.05) hatchability, hatchability of fertile eggs, or the duration of the hatch window. The chick hatching distribution was uniform and began after 456 h of incubation, regardless of breeder age (Table 2; Figure 1).

**Table 2:** Hatchability, hatch of fertile eggs, and hatch window duration (h) of broiler chicks from heavy breeder hens of different ages.

Breeder age (weeks)	Hatchability (%)	Hatch of fertile eggs (%)	Hatch window duration (h)
39	78.45	82.06	27
51	75.93	76.73	29
69	67.59	71.94	30
*p* value	0.268	0.293	0.705
SEM	4.816	4.455	2.556

Abbreviation: SEM, standard error of the mean.

Breeder age significantly affected (*p* < 0.05) egg and chick characteristics. Eggs from 51‐ and 69‐week‐old breeders presented greater weights than those from 39‐week‐old breeders did. Chicks from these breeders also presented greater gross and net body weights and longer body lengths at hatch. However, for gross and net body weights, chicks from 51‐week‐old breeders did not differ from those derived from 39‐week‐old breeders. Breeder age also influenced progeny traits, with chicks from 69‐week‐old breeders presenting greater body weights at hatch and at placement. In addition, chicks from 51‐ and 69‐week‐old breeders presented lower dehydration rates. No effect of breeder age was observed (*p* > 0.05) for the remaining variables (Table 3).

**Table 3:** Egg and chick characteristics during incubation and at hatch from heavy broiler breeders of different ages.

Variables	Breeder age (weeks)			*p* value	SEM
	39	51	69		
Egg weight (EW) (g)	65.47^b^	70.22^a^	70.66^a^	< 0.001	0.857
Chick weight with yolk sac (g)	43.78^b^	45.97^ab^	47.27^a^	0.004	0.726
Chick length (cm)	17.52^b^	18.28^a^	18.46^a^	< 0.001	0.080
Yolk sac weight (g)	6.30	6.54	6.81	0.700	0.426
Chick net weight (g)	37.46^b^	39.43^ab^	40.45^a^	0.004	0.628
Chick in egg (%)	57.26	56.17	57.27	0.406	0.658
Yolk sac (%)	14.35	14.04	14.34	0.958	0.848
EW loss during incubation (%)	13.02	14.76	13.53	0.129	0.615
Hatch weight (g)	47.48^c^	49.10^b^	51.88^a^	< 0.001	0.355
Pull‐out weight (g)	44.08^c^	46.34^b^	48.64^a^	< 0.001	0.360
Chick dehydration (%)	7.14^a^	5.63^b^	6.27^b^	0.001	0.332

*Note:* Means followed by different superscript letters within a row differ by Tukey's test (*p* < 0.05).

Abbreviation: SEM, standard error of the mean.

Embryonic mortality was significantly affected (*p* < 0.05) by breeder age during the early stage of embryonic development (Phase I), with embryos from 69‐week‐old breeders showing higher mortality rates. No significant differences (*p* > 0.05) in embryonic mortality were detected during the subsequent developmental phases (Table 4).

**Table 4:** Embryodiagnosis of nonhatched eggs from heavy breeder hens of different ages.

Diagnosis (%)	Breeder age (weeks)			*p* value
	39	51	69	
Infertility	3.77	1.48	6.67	0.298
Phase I	4.11^b^	5.20^b^	11.82^a^	0.007
Phase II	0.37	1.85	1.12	0.443
Phase III	3.36	1.49	3.40	0.301
Phase IV	3.74	7.43	2.96	0.428
Dead pip	2.23	4.69	3.36	0.802
Live pip	1.89	0.75	0.77	0.960
Contaminated	1.88	4.20	2.64	0.609

*Note:* Means followed by different superscript letters within a row differ significantly (*p* < 0.05) according to Dunn's test following the Kruskal‐Wallis test.

Embryonic development was influenced by breeder age at specific stages of incubation. Embryos from younger breeders presented a greater relative proportion in relation to egg size on days 6 and 13 of incubation (*p* < 0.05). However, considering absolute embryo weight, embryos from 69‐week‐old breeders were heavier than those from 51‐week‐old breeders were on day 13 of incubation (*p* < 0.05). At 18 days of incubation, the embryos from 69‐week‐old breeders also presented greater absolute weights. Breeder age did not affect (*p* > 0.05) liver weight (Table 5).

**Table 5:** Relative embryo weight in relation to egg weight and relative liver weight in relation to embryo weight of broilers from heavy breeder hens of different ages during embryonic development and at hatch.

Variables		Breeder age (weeks)			*p* value	SEM
		39	51	69		
Relative embryo weight (%)	6° day	1.61^a^	1.51^ab^	1.28^b^	0.015	0.078
	13° day	23.01^a^	21.29^b^	20.69^b^	< 0.001	0.367
	18° day	48.15	50.20	49.10	0.378	1.029
Embryo weight (g)	6° day	1.07	1.05	0.90	0.060	0.050
	13° day	14.83^ab^	14.43^b^	15.41^a^	0.016	0.233
	18° day	31.99^b^	34.04^b^	37.07^a^	< 0.001	0.779
Relative liver weight (%)	13° day	1.50	1.61	1.41	0.588	0.141
	18° day	1.53	1.58	1.53	0.462	0.086
	Neonate	2.28	2.53	2.45	0.358	0.123

*Note:* Means followed by different superscript letters within rows differ by Tukey's test (*p* < 0.05).

Abbreviation: SEM, standard error of the mean.

Breeder age affected chick physical quality scores. Chicks from 51‑ and 69‑week‑old breeders had a greater frequency of scores between 86 and 90 (Table 6).

**Table 6:** Physical quality score (0 to 100 points*) of newly hatched chicks from heavy breeder hens of different ages.

Score (%)	Breeder age (weeks)			*p* value
	39	51	69	
< 70	13.23	3.40	7.69	0.423
71–75	1.59	0.56	0.00	0.312
76–80	9.53	6.11	4.78	0.559
81–85	7.14	0.00	0.00	0.101
86–90	24.88^b^	47.38^a^	53.39^a^	0.050
91–95	8.77	14.90	4.74	0.336
96–100	30.13	27.33	20.27	0.502

*Note:* *Score adapted from Tona et al. (2003). Means followed by different superscript letters within a row differ by the Kruskal–Wallis test (*p* < 0.05).

The eggshell calcium content did not differ (*p* > 0.05) among breeders at any incubation time point (Table 7).

**Table 7:** Eggshell calcium content of preincubation eggs from heavy breeder hens of different ages.

Breeder age (weeks)		Incubation period (days)			
		0	13°	18°	21°
39	Calcium (g. kg⁻¹)	306.63	244.39	209.63	200.04
51		300.26	232.21	204.94	197.13
69		297.16	235.38	210.85	198.51
*p* value		0.285	0.316	0.389	0.549
SEM		4.132	5.514	3.047	1.820

Abbreviation: SEM, standard error of the mean.

The serum glucose concentrations of neonatal chicks did not differ among the treatments (*p* > 0.05), regardless of hatching time (Table 8). However, the plasma glucose levels increased linearly with hatching time, as described by the regression equation *y* = −55.569340 + 0.501841*x* (*R* ^2^ = 0.16; *p* = 0.005).

**Table 8:** Serum glucose levels of chicks from heavy breeder hens of different ages according to hatching time.

	Glucose (mg/dL)
Breeder age (weeks)	
39	192.72
51	186.33
69	186.86
Hatching time (h)	
0	180.00
12	185.32
24	189.21
36	200.04
*p* value	
Age (A)	0.4504
Time (T)	0.0428
A x T	0.3704
CV (%)	8.24

Abbreviation: CV, coefficient of variation.

**Figure 2:**
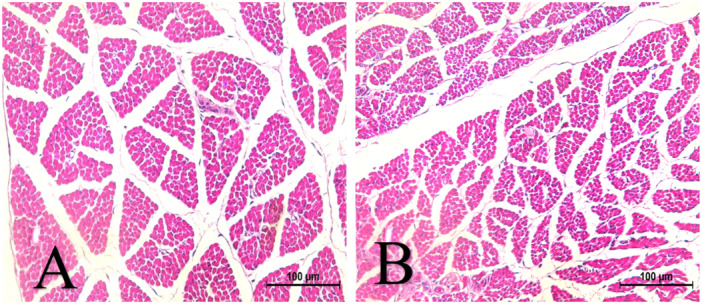
Representative histological sections of the pectoralis major muscle from one‐day‐old female (A) and male (B) broiler chicks used for muscle fiberz counting. [Color figure can be viewed at wileyonlinelibrary.com]

Finally, the number of breast muscle fibers in newly hatched female chicks was significantly affected (*p* < 0.05) by breeder age. Chicks from older breeders presented a greater number of muscle fibers, suggesting enhanced muscle growth potential. In contrast, no significant differences (*p* > 0.05) were detected for male chicks or for other muscle development variables among the treatments (Table 9; Figure 2).

**Table 9:** Number of breast muscle fibers of newly hatched male and female chicks from heavy breeder hens of different ages.

Breeder age (weeks)		
	Male	Female
39	54.8	45.8^b^
51	62.4	67.2^a^
69	60.8	60.8^a^
*p* value	0.429	0.006
SEM	4.201	3.934

*Note:* Means followed by different superscript letters within a column differ by Tukey's test (*p* < 0.05).

Abbreviation: SEM, standard error of the mean.

## 4 Discussion

Egg weight plays a crucial role in incubation performance, directly affecting weight loss during incubation, embryonic mortality, chick production, and final body weight (Duman and Şekeroğlu 2017). Breeder age has been consistently associated with changes in egg characteristics, particularly increases in egg weight as hens age, as reported by Damasceno et al. (2017) and Araújo et al. (2016).

As breeder hens age, the ovulation interval increases, resulting in a reduction in the laying rate but an increase in egg weight. This increase is associated with a reduction in the number of developing follicles, causing a similar amount of yolk synthesized by the liver to be deposited into larger follicles, thereby increasing the yolk proportion within the egg (Burnham et al. 2001). Consequently, older breeders not only produce larger eggs but also increase the yolk–egg weight ratio, favoring nutrient transfer to the embryo (Peebles et al. 2001).

In addition, the size of internal egg components, such as yolk and albumen, directly affects the amount of nutrients available to the embryo and its capacity to absorb them (Yadgary et al. 2013). Differences in nutrient availability and utilization efficiency during embryogenesis have also been reported across distinct breeder genotypes (Onbaşılar et al. 2017). Breeder age influences the size of these components, with eggs from older breeders showing increases in both yolk and albumen contents (Garcia et al. 2010; Nangsuay et al. 2016). These changes also modify egg chemical composition, as eggs from younger breeders tend to contain lower quantities of these components, which may limit early embryonic development (Peebles et al. 2001; Vieira et al. 2005; Lourens et al. 2006). The yolk, as the primary nutrient source for the embryo, contains lipids, proteins, vitamins, and minerals, and its contents begin to be assimilated from the second day of incubation onward, when the yolk sac is formed (Bauer et al. 2013; Yadgary et al. 2014). Therefore, eggs with greater yolk and albumen contents provide higher levels of essential nutrients, promoting more robust embryonic development.

Another critical factor in embryonic development is eggshell quality, particularly shell thickness, porosity, and specific gravity. Broiler breeder age can significantly affect shell quality, thereby directly influencing incubation success (Nowaczewski et al. 2016). Vilela et al. (2016) reported that as laying hens age, shell thickness decreases and may be associated with increased shell porosity, which compromises embryonic performance during incubation.

Consistent with these findings, Dias et al. (2025) suggested that the increase in egg size associated with breeder ageing reduces shell quality, as egg weight increases more rapidly than shell volume does, resulting in a lower shell proportion in eggs from older breeders. Reduced shell thickness in breeding flocks of advanced age has been widely attributed to age‑related inefficiencies in calcium metabolism and shell deposition.

The decline in shell quality with increasing breeder age may be attributed to a reduced intestinal calcium absorption capacity and decreased mobilization of calcium from medullary bone, compounded by the larger egg size in older hens, which results in greater calcium deposition occurring with lower efficiency (Nys et al. 2004). Similar age‑related changes in yolk mineral dynamics and embryonic development have been described in other avian species without consistent impairment of hatchability (Onbaşılar et al. 2014). In addition, advancing age has been associated with increased production of reactive oxygen species, which may impair bone metabolism by inhibiting osteoblast differentiation and proliferation while stimulating osteoclastogenesis, thereby further limiting calcium availability for eggshell formation (Ming et al. 2009; Oliveira et al. 2021). In younger breeders, higher efficiency of calcium utilization for shell mineralization results in eggs with a higher shell percentage (Vilela et al. 2016). These findings are consistent with those of Camargo et al. (2022), who reported reduced shell thickness in eggs from older commercial laying hens.

In addition to shell quality, the quality of internal egg components is also a determining factor for embryonic development. The yolk and albumen proportions (yolk and albumen indices) are important indicators of internal egg quality, and as observed in this study, breeder age influences these indices. The increase in egg and yolk weight in older breeders, accompanied by a reduction in internal quality, reflects increased water transfer from albumen to yolk, which may negatively affect yolk quality and compromise embryonic development (Silversides and Scott 2001; Jones and Musgrove 2005).

Eggshell quality also directly affects embryonic mortality, particularly during early incubation stages. Yamak et al. (2023) and Kroetz Neto et al. (2024) reported recent evidence that eggs with lower specific gravity lose more moisture, exhibit higher early embryonic mortality, and hatch less successfully; concurrently, breeder aging is linked to a reduced shell proportion/quality (thinner, more porous shells), making these eggs more susceptible to dehydration and impaired embryonic development. This pattern has been associated with chick dehydration at hatch, particularly in progeny from older breeder flocks, likely due to greater yolk reserves and higher hatch weight associated with older flocks.

Previous studies have reported divergent effects of breeder age on hatchability and hatch of fertile eggs. Araújo et al. (2016), when evaluating different incubation systems, observed lower hatchability in eggs from older breeders. In contrast, Machado et al. (2020) found no significant effect of breeder age on hatchability in slow‐growing broilers, suggesting that incubation conditions and genotype may modulate age‐related effects. Embryodiagnosis data further indicate that when mortality occurs in eggs from older breeders, it is predominantly concentrated at early embryonic stages, a pattern commonly associated with increased moisture loss during incubation. The lack of a statistically significant effect of breeder age on hatchability in this study likely reflects the experimental scale and controlled incubation conditions, which differ from large‑scale commercial hatchery environments where cumulative age‑related effects are more pronounced.

The results obtained in this study, in which embryos from younger breeders presented greater relative weights during the early incubation period, are in agreement with the findings of Peebles et al. (2001), who evaluated the effects of breeder age and dietary fat sources and levels on embryonic development in broilers. Chick weight at hatch is an important quality indicator. Iqbal et al. (2016) reported a well‐defined positive correlation between egg size and chick body weight at hatch. Similarly, Vieira et al. (2005) reported that chicks derived from large eggs laid by 40‐week‐old Ross breeders had greater hatch weights than chicks from smaller eggs.

However, evaluation of embryonic development revealed an opposite trend, in which embryos from younger breeders exhibited greater relative yield and higher relative weight in relation to egg size. This phenomenon may be explained by the nutritional characteristics of eggs from younger breeders, which are smaller but contain denser and more concentrated albumen and yolk, providing embryos with a greater nutrient supply during early development. Albumen, as the main nutrient source at this stage, is preferentially utilised by the embryo (Nangsuay et al. 2016).

With respect to liver development and other organs critical for metabolism, Lana et al. (2000) reported that the increased metabolic rate of modern broilers, driven by rapid genetic growth, requires greater nutrient availability. Gluconeogenesis and glycogenesis intensify around day 17 of incubation, explaining the increased nutritional demand. Although eggs from older breeders contain greater yolk lipid content and are expected to promote greater liver development, Ulmer‐Franco et al. (2010) reported delayed embryonic growth in eggs from younger breeders due to lower nutrient availability and slower absorption rates in smaller eggs.

Conversely, the findings of the present study differ from those of Zocche et al. (2016), who reported greater relative liver weight in embryos from older breeders. These discrepancies suggest that liver development may also be influenced by other factors, such as albumen quality and incubation conditions.

Environmental factors play a critical role in embryonic development. Lourens et al. (2006) and Hamidu et al. (2007) reported differences in metabolism and oxygen consumption between embryos from younger (< 35 weeks) and older (> 50 weeks) breeders. The incubation temperature, particularly during the final week, may negatively affect embryonic development and posthatch performance (Lourens et al. 2006), highlighting the importance of fine‐tuning environmental conditions during incubation.

Breeder age clearly influences both egg quality and embryonic development. Older breeders produce heavier eggs, a pattern consistently associated with increased yolk deposition and enhanced nutrient availability to the developing embryo. However, age‐related changes in egg structure result in a trade‐off between nutrient supply and incubation efficiency, as structural limitations of the egg can affect the maintenance of optimal incubation conditions.

The incubation environment plays a determinant role in modulating these age‑related effects, particularly through appropriate control of temperature and humidity. The chick quality outcomes observed align with the scoring framework proposed by Tona et al. (2004), reinforcing the close association among breeder age, egg structural characteristics, and hatchling quality. Collectively, the evidence indicates that although breeder age alters key egg and incubation traits, adequate incubation management can support proper embryonic development and the production of high‑quality chicks.

The eggshell mineral content was also evaluated. The eggshell represents an important mineral reservoir that is essential for embryonic skeletal formation. Although Vilela et al. (2016) reported that older hens produce eggs with lower shell calcium concentrations, Fu et al. (2024) reported that total calcium deposition throughout the laying cycle remains relatively constant, supporting continuous mineral transfer to the egg without compromising embryonic development.

Serum glucose levels, which reflect embryonic metabolic status, were also assessed. Borges et al. (2021) reported that blood glucose concentrations in broilers range from 200 to 500 mg/dL. The values observed in the present study were within this normal physiological range. The difference observed between late‐ and early‐hatching chicks may be explained by lactate recycling into glucose, a metabolic adaptation that ensures a continuous energy supply during the immediate posthatching period (Van De Ven et al. 2011). Finally, the variation in glucose levels between male and female chicks may be attributed to hormonal or genetic factors, as described by Scanes (2011).

Muscle fiber formation during embryogenesis is a key determinant of post‐hatch muscle growth and meat production in broilers since the total number of muscle fibers is established before hatch. This process depends on the proliferation and differentiation of myoblasts during embryonic development, which can be influenced by maternal factors such as nutrient availability in the egg and incubation conditions. Variations in egg composition, particularly in the supply of lipids and proteins derived from the yolk and albumen, may affect embryonic myogenesis and consequently the development of the *pectoralis major* muscle. In this context, maternal characteristics and egg nutrient deposition may play important roles in regulating muscle fiber formation and the growth potential of broiler progeny (Rehfeldt et al. 2000).

## 5 Conclusion

Breeder age influenced egg characteristics, incubation responses, and early progeny development. Advanced breeder age favoured egg size, chick weight, and physical quality at hatch, whereas reduced eggshell integrity increased early embryonic vulnerability. These findings indicate a biological trade‑off in which gains in egg and chick size with breeder ageing are accompanied by limitations in shell quality and early viability, emphasising the need to consider breeder age when managing incubation strategies for heavy breeders.

## Author Contributions

**Helder Freitas de Oliveira:** conceptualization, investigation, data curation, visualization, writing – original draft, writing – review and editing. **Alessandra Gimenez Mascarenhas:** methodology, investigation, validation. **José Henrique Stringhini:** conceptualization, supervision, and funding acquisition. **Nadja Susana Mogyca Leandro:** supervision, project administration. **Marcos Barcellos Cafe:** methodology, resources. **Emmanuel Arnhold:** statistical analysis, validation. **Raíssa Monteiro de Alvarenga:** investigation, data curation. **Ana Caroline Romão Silva:** investigation. **Jean Kaique Valentim:** writing – review and editing. **Heloisa Helena de Carvalho Mello:** conceptualization, supervision, funding acquisition. All the authors read and approved the final version of the manuscript.

## Funding

The authors have nothing to report.

## Ethics Statement

The animal study protocol was approved by the Ethics Committee on Animal Use of the Federal University of Goiás (CEUA/UFG protocol No. 076/18). All procedures were conducted in accordance with the Brazilian National Council for the Control of Animal Experimentation (CONCEA) guidelines and the ARRIVE guidelines.

## Conflicts of Interest

The authors declare no conflicts of interest.

## Data Availability

The data that support the findings of this study are available from the corresponding author upon reasonable request.
